# Modifying homes for persons with physical disabilities in Thailand

**DOI:** 10.2471/BLT.16.178434

**Published:** 2017-02-01

**Authors:** Sirinart Tongsiri, Chanuttha Ploylearmsang, Katanyu Hawsutisima, Wachara Riewpaiboon, Viroj Tangcharoensathien

**Affiliations:** a Quality of Life Research Unit, Faculty of Medicine, Mahasarakham University, Nakornsawan Road, Tambon Talad, Muang, Mahasarakham 44000, Thailand.; b Social Pharmacy Research Unit, Faculty of Pharmacy, Mahasarakham University, Mahasarakham, Thailand.; c Faculty of Architecture, Urban Design and Creative Arts, Mahasarakham University, Mahasarakham, Thailand.; d Health System Research Institute, Nonthaburi, Thailand.; e International Health Policy Program, Ministry of Public Health, Nonthaburi, Thailand.

## Abstract

**Problem:**

Thailand passed the Persons with Disabilities Empowerment Act in 2007. The Act, which is in compliance with the United Nations *Convention on the Rights of Persons with Disabilities*, ensures that registered persons with disabilities are entitled to home environment modifications’ benefits up to a maximum of 20 000 baht (670 United States dollars); however, the Act’s enforcement is still weak in Thailand.

**Approach:**

In 2013, researchers developed a home modification programme, consisting of a multidisciplinary team of medical and nonmedical practitioners and volunteers, to modify homes for persons with disabilities. The programme recruited participants with physical disabilities and assessed their functioning difficulties. Participants’ homes were modified to address identified functioning difficulties.

**Local setting:**

The project was implemented in four provinces in collaboration with staff from 27 district hospitals located in north-eastern Thailand.

**Relevant changes:**

After the home modifications, all 43 recruited participants reported reduced difficulties in all areas, except for participants with severe degrees of difficulties, such as those reporting being unable to walk and unable to get up from the floor. The participants’ quality of life had also improved. The average EQ-5D-5L score, measuring quality of life, increased by 0.203 – from 0.346 at baseline to 0.549 after the modifications.

**Lessons learnt:**

Home modifications in low-resourced settings are technically and financially feasible and can lead to reducing functioning difficulties and improving the quality of life of persons with disabilities. Implementation requires government subsidies to finance home modifications and the availability of technical guidelines and training on home modifications for implementing agents.

## Résumé

**Problème:**

En 2007, la Thaïlande a promulgué une loi sur l'autonomisation des personnes vivant avec un handicap. Cette loi, conforme à la Convention des Nations Unies relative aux droits des personnes handicapées, stipule que les personnes handicapées recensées ont le droit de bénéficier d’une modification de leur domicile, à hauteur de 20 000 bahts maximum (670 dollars des États-Unis). Or, l'application de la cette loi reste limitée en Thaïlande.

**Approche:**

En 2013, des chercheurs ont conçu un programme de modification du domicile, consistant à faire intervenir une équipe multidisciplinaire de volontaires et de professionnels médicaux et non-médicaux afin d'adapter le domicile de personnes handicapées. Dans le cadre de ce programme, plusieurs participants présentant un handicap physique ont été recrutés, et leurs difficultés dans la vie quotidienne ont été évaluées. Les domiciles des participants ont ensuite été adaptés en fonction des difficultés du quotidien ainsi identifiées.

**Environnement local:**

Ce projet a été mis en œuvre dans quatre provinces, en collaboration avec le personnel de 27 hôpitaux de districts du Nord-Est de la Thaïlande.

**Changements significatifs:**

Après modification de leur domicile, les 43 participants recrutés ont tous affirmé rencontrer moins de difficultés dans tous les domaines, excepté pour les participants confrontés à des difficultés particulièrement lourdes, tels que ceux qui avaient indiqué être incapables de marcher et de se relever du sol. La qualité de vie des participants s'est également améliorée. Le score moyen obtenu avec le questionnaire EQ-5D-5L (qui évalue la qualité de vie) a augmenté de 0,203, en passant de 0,346 au début du programme à 0,549 après modification des domiciles.

**Leçons tirées:**

La modification des domiciles dans des contextes de faibles ressources est techniquement et financièrement réalisable et peut entraîner une réduction des difficultés rencontrées par les personnes handicapées dans leur vie quotidienne et améliorer leur qualité de vie. Pour la réalisation d'un tel projet, des subventions gouvernementales sont nécessaires, et les intervenants chargés de la mise en œuvre doivent pouvoir accéder à des directives techniques et à une formation sur la modification des domiciles.

## Resumen

**Problema:**

En 2007, Tailandia aprobó la Ley para el Empoderamiento de las Personas con Discapacidad. La Ley, que cumple con la Convención de las Naciones Unidas sobre los Derechos de las Personas con Discapacidad, garantiza que las personas registradas con discapacidad tienen derecho a adaptaciones del entorno del hogar hasta un máximo de 20 000 baht (670 dólares estadounidenses). No obstante, la aplicación de la Ley sigue siendo débil en Tailandia.

**Enfoque:**

En 2013, investigadores desarrollaron un programa de adaptación de hogares, formado por un equipo multidisciplinar de practicantes médicos y de otras especialidades y voluntarios, para adaptar los hogares de las personas con discapacidad. El programa captó participantes con discapacidades físicas y evaluó sus dificultades de funcionamiento. Se adaptaron los hogares de los participantes para atender las dificultades de funcionamiento identificadas.

**Marco regional:**

El proyecto se implementó en cuatro provincias, en colaboración con el personal de 27 hospitales de distrito localizados en el noreste de Tailandia.

**Cambios importantes:**

Tras realizar las adaptaciones de los hogares, los 43 participantes implicados informaron de la reducción de las dificultades en todas las zonas, salvo para los participantes con altos grados de dificultad, como los que no podían andar ni levantarse del suelo. También mejoró la calidad de vida de los participantes. La puntuación media del EQ-5D-5L, que mide la calidad de vida, aumentó un 0,203: de un 0,346 al inicio a un 0,549 tras las adaptaciones.

**Lecciones aprendidas:**

Las adaptaciones de los hogares en regiones de escasos recursos son factibles a nivel técnico y económico, y pueden reducir las dificultades de funcionamiento, así como mejorar la calidad de vida de las personas con discapacidad. La implementación requiere subvenciones del gobierno para financiar las adaptaciones de los hogares y la disponibilidad de directrices y formación a nivel técnico sobre las adaptaciones de los hogares para los agentes que realizan la implementación.

## ملخص

**المشكلة:**

أقرت تايلند قانون تمكين الأشخاص من ذوي الإعاقات في عام 2007. ويأتي هذا القانون الذي يتوافق مع اتفاقية الأمم المتحدة حول حقوق الأشخاص من ذوي الإعاقات ليضمن أحقية حصول الأشخاص من ذوي الإعاقات المسجلين على مزايا إجراء التعديلات في بيئة المنزل بمبلغ يصل إلى 20 ألف بات (670 دولارًا أمريكيًا)؛ ومع ذلك، لا تزال إجراءات تطبيق القانون ضعيفة في تايلند.

**الأسلوب:**

قام الباحثون في عام 2013 بإعداد برنامج لتعديل المنازل، وهو يتكون من فريق متعدد التخصصات يتألف من أطباء ممارسين وأطباء غير ممارسين ومتطوعين، بغرض التعديل على المنازل لتكون ملائمة للأشخاص من ذوي الإعاقات. ضم البرنامج مشاركين من ذوي الإعاقات البدنية وعمل على تقييم صعوبات أداء وظائفهم البدنية. وتم تعديل منازل المشاركين لمعالجة صعوبات أداء الوظائف البدنية المحددة.

**المواقع المحلية:**

تم تنفيذ المشروع في أربع مقاطعات، بالتعاون مع طاقم العاملين من 27 من مستشفيات المحافظات الموجودة في شمال شرق تايلند.

**التغييرات ذات الصلة:**

بعد إجراء التعديلات على المنازل، أبلغ جميع المشاركين المنضمين البالغ عددهم 43 عن انخفاض درجة الصعوبة في جميع المناطق، باستثناء المشاركين الذين يواجهون درجات صعوبة شديدة، على سبيل المثال أولئك الأشخاص الذي يبلغون عن عدم قدرتهم على المشي وعدم قدرتهم على النهوض من على الأرض. كما تحسنت أيضًا نوعية الحياة التي يعيشها المشاركون. ولقد زاد معدل EQ-5D-5L المتوسط الذي يهدف لقياس نوعية الحياة بمقدار 0.203 من 0.346 عند خط الأساس إلى 0.549 بعد إجراء التعديلات.

**الدروس المستفادة:**

إن إجراء تعديلات على المنازل في المناطق التي تفتقر إلى الموارد ممكن الحدوث من الناحية الفنية والمالية ويمكن أن يؤدي إلى تقليل صعوبات أداء الوظائف البدنية وتحسين نوعية الحياة التي يعيشها الأشخاص من ذوي الإعاقات. يتطلب تنفيذ هذه الإجراءات منح المساعدات الحكومية لتمويل التعديلات على المنازل وتوفير الإرشادات الفنية والتدريب الخاص بإجراء التعديلات على المنازل للأشخاص المكلفين بالتنفيذ.

## 摘要

**问题:**

泰国通过了《2007 年残疾人赋权法》。该法案根据《联合国残疾人权利公约》的规定，确保登记在册的残疾人士有权享受最高可达 20 000 泰铢（670 美元）的住宅环境改造福利；但该法案在泰国的执行力度仍很薄弱。

**方法:**

2013 年，研究人员发起了一项住宅改造项目，由医疗和非医疗从业人员及志愿者组成了一个多学科团队，为残疾人士进行住宅改造。该项目招募了患有身体残疾的参与者，并评估了他们的机能障碍。对参与者的住宅设施进行了改造，以解决已发现的机能障碍。

**当地状况:**

该项目在四个省份实施，与来自泰国东北部 27 个地区医院的员工展开了合作。

**相关变化:**

住宅改造之后，除具有重度障碍的参与者（例如报告显示无法行走和无法起身）之外，43 位招募的参与者均报告在各方面的障碍都有所减少。参与者的生活品质也得到了提升。衡量生活品质的 EQ-5D-5L 平均得分提高了 0.203——从基准线的 0.346 提高到改造后的 0.549。

**经验教训:**

在资源稀缺的情况下进行住宅改造从技术上和经济上都具有可行性，并且可减少残疾人的机能障碍和提升其生活品质。住宅改造项目的实施需要政府提供资金补贴，并为实施机构提供住宅改造技术指导和培训。

## Резюме

**Проблема:**

В 2007 году в Таиланде был принят закон «О расширении прав и возможностей людей с инвалидностью». Закон, принятый в соответствии с Конвенцией о правах инвалидов Организации объединенных наций, гарантирует, что поставленные на учет лица с инвалидностью имеют право на выплаты для изменения жилищных условий размером до 20 000 батов (670 долларов США); тем не менее этот закон до сих пор слабо претворяется в жизнь в Таиланде.

**Подход:**

В 2013 году многодисциплинарная группа исследователей, включающая работников как медицинской, так и немедицинской сфер, а также добровольцев, разработали программу изменения жилищных условий для изменения жилищных условий людей с инвалидностью. В соответствии с программой был проведен набор участников с физическими недостатками и были определены ограничения их жизнедеятельности. Жилища участников были изменены для уменьшения определенных ограничений жизнедеятельности.

**Местные условия:**

Проект был реализован в четырех провинциях при поддержке персонала из 27 районных больниц, расположенных в северо-восточном Таиланде.

**Осуществленные перемены:**

После внесения изменений в жилищные условия все 43 отобранных участника сообщили об уменьшении ограничений во всех сферах, за исключением тех участников, ограничения в жизнедеятельности которых были значительны. К ним относятся, например, участники, сообщившие, что они неспособны ходить или неспособны подняться с пола. Качество жизни участников также повысилось. Средний результат оценки с помощью опросника EQ-5D-5L, предназначенного для измерения качества жизни, увеличился на 0,203: с 0,346 (исходные данные) до 0,549 (после изменений).

**Выводы:**

Изменения жилищных условий в рамках ограниченности ресурсов целесообразны с технической и финансовой точки зрения и могут способствовать уменьшению ограничений в жизнедеятельности и повышению качества жизни людей с инвалидностью. Для реализации такого проекта необходимы правительственные субсидии для финансирования изменений жилищ и наличие технических руководств по способам изменения жилищных условий и соответствующих курсов подготовки для людей, ответственных за реализацию проекта.

## Introduction

Home environment modifications are essential to improve the quality of life of persons with disabilities. Parts of a home and its surroundings, together with the built environment should be changed according to the impairments of persons with disabilities to minimize difficulties in activities of daily living and to alleviate the burden on carers. Article 28 of the United Nations *Convention on the Rights of Persons with Disabilities* ratified by Thailand in 2008 endorsed the right of persons with disabilities to independent living.^1^ International experiences show different countries use more than one funding mechanism to finance home environment modifications that aim to enhance the independent living of persons with disabilities.^2^^–^^4^ A regulation promulgated by the Thai Ministry of Social Development and Human Security describes the appropriate home surroundings and built environment to be accessible by persons with disabilities,^5^ however the enforcement of the regulation is still weak.

## Local setting

Thailand is an upper middle-income country with an estimated population of 68 million in 2015.^6^ Thailand passed the Persons with Disabilities Empowerment Act in 2007. The Act which is in compliance with the United Nations *Convention on the Rights of Persons with Disabilities*, ensures that registered persons with disabilities are entitled to government subsidies for home environment modification to a maximum of 20 000 Thai baht (equivalent to 670 United States dollars).^7^ Provincial social development and human security offices are responsible for implementing this benefit. In addition, the provincial rehabilitation fund, which has been set up by a 50–50 contribution from the Thailand National Health Security Office and the Provincial Administration Organization, also provides financial support for home modifications to persons with disabilities.

## Approach

In 2013, researchers from Mahasarakham University, faculty of medicine, developed a home environment modification programme for persons with physical disabilities. The researchers formed a project team consisting of two architects, two engineers, a group of local builders, nurses and social workers from communities where the selected homes were located. The project was implemented in four provinces Kalasin, Khonkaen, Mahasarakham and Roi-Et, in collaboration with 27 district hospitals located in the provinces. These hospitals have health-care teams, consisting of physical therapists, nurses and health care volunteers who visit persons with disabilities in their homes. We asked these teams to recruit persons with physical disabilities, including elderly people, residing in these provinces to participate in the programme. Researchers from the faculty of medicine ran four training sessions for the project team on universal design concept application and on how to do home and built-environment modifications in order to enhance the functions of persons with disabilities.

## Functioning difficulty assessment

The physical therapists and primary care nurses working in the four district hospitals identified, selected and clinically examined eligible participants in their homes and asked the participants to sign the consent form. The project team set the participants’ selection criteria, based on the type of functioning difficulties the identified person with disabilities had. Functioning was measured using the *International classification of functioning, disability and health* (ICF).^8^^,^^9^ Seven difficulty levels as defined in the ICF were used as selection criteria: (i) having difficulties walking (ICF code d450); (ii) having difficulties getting up from the floor (d4101); (iii) having difficulties getting up from a chair or bed (d4103); (iv) having difficulties moving around (d460); (v) having difficulties climbing (d4551); (vi) moving around using equipment (d465); (vii) having at least some difficulties walking (d450) with assistive devices or with assistive devices and personal support. Quality of life and risk from fall were measured using EQ-5D-5L^10^ and the Berg Balance Scale Test,^11^ respectively. EQ-5D-5L is a standardized instrument used to measure quality of life using five dimensions: mobility, self-care, usual activities, pain and discomfort, and anxiety and depression.^10^ The Berg Balance Scale Test is used to monitor dynamics and static balance abilities to predict fall risk.^11^ The other criteria for participant selection were home ownership and willingness to participate in the modification programme. The researchers visited the homes identified for modification to document the existing structure and the surrounding neighbourhood. The researchers took photographs and short video clips that show the general condition of the homes and parts of the homes to be modified and the daily activities performed by the persons with disabilities residing in the homes. The physical therapists and primary care nurses reassessed the participants’ functioning difficulties after the home modifications were completed through assessments in the participants’ homes.

## Home modifications

We assigned the selected homes into four groups according to the type of modifications needed. Group 1 included those homes that required minimal changes, such as installing handrails or building wider doors. Group 2 included those homes that required changing some part of the home, for example, relocating a toilet to be closer to the participant’s living area. In Group 3 modifications weren’t possible in the homes for various reasons, therefore newer quarters needed to be built. In Group 4 home modifications were necessary, but only to ensure safety and security for the participant, rather than to improve the enabling environment, for example replacing a new roof or filling a specific piece of land to prevent flooding. The number of homes in each group, the cost of modifications and the duration of the construction for each group is presented in Table 1.

**Table 1:** Quality of life scores before and after home modifications, Thailand, 2013

Type of home modifications	No. (%) of homes	Duration of construction, days	Average cost of modifications, US$	EQ-5D-5L baseline score	EQ-5D-5L after modifications score	Net score change
Group 1: Modifications with minimum changes	12 (29)	2–15	697	0.337	0.495	0.158
Group 2: Modifications in some part of the home	20 (47)	3–20	1384	0.410	0.574	0.164
Group 3: New quarters built	9 (20)	11–30	2130	0.198	0.546	0.348
Group 4: Modifications to ensure safety and security	2 (4)	5 and 14	744	0.184	0.511	0.327
All	**43 (100)**	**NA**	**NA**	**0.346**	**0.549**	**0.203**

NA: not applicable; US$: United States dollars.

Note: EQ-5D-5L is a standardized instrument measuring quality of life, using five dimensions: mobility, self-care, usual activities, pain and discomfort and, anxiety and depression. Each dimension contains an additional five levels: no problem, slight problem, moderate problem, severe problem and extreme problem.^10^ EQ-5D-5L scores range from −1 to 1, with −1 being the lowest quality of life score and 1 being the highest quality of life score.

## Financing the modifications

In addition to the government subsidies, provincial rehabilitation and subdistrict administrative organizations’ funds and donations from participants’ families were used to pay for home modifications. Volunteers from the local community, for example, family members, Buddhist monks, soldiers and other villagers worked on the modifications. In the areas where volunteers were unavailable, local professional builders were hired. On-site coordination and management were done by district hospital staff. The government subsidies and the provincial rehabilitation funds covered approximately 70% of the modification costs, and were used mostly to buy building material. The remaining 30% was mobilized from subdistrict administrative organizations and households and was used to cover labour cost, including meals for volunteers.

## Relevant changes

Of the 77 persons with disabilities we identified, we recruited 62 persons to participate in the project. Six participants died before the home modifications started, two others died later during the modifications and eleven were still waiting for funding when the project started. Therefore, only 43 participants were included in the project and their 43 homes were successfully modified.

The level of difficulty experienced by the participants when performing daily activities was assessed before and after the home modifications. When compared with the baseline assessment, this had decreased after the modifications. At the baseline assessment, the most frequently reported activities performed with difficulties by the 43 participants were, walking 97.7% (42), getting up from the floor 88.4% (38), getting up from a chair/bed 62.8% (27), moving around inside the home 30.2% (13), moving around outside the home using equipment 39.5% (17) and climbing stairs 23.3% (10). After the modifications, the level of difficulties decreased for 23.8% (10/42) of the participants who had reported having difficulties walking and 29.6% (8/27) of those who had reported having difficulties getting up from a chair/bed. The decrease for other activities was 44.7% (17/38) for getting up from the floor, 38.5% (5/13) for moving around inside the home and 11.8% (2/17) for moving around outside the home using equipment. As shown in Table 2, after the home modifications, the number of participants reporting that their difficulties were reduced had increased in all function areas except for participants with severe degrees of difficulties, such as those reporting themselves as unable to walk (d450) and unable to get up from the floor (d4101), indicating that home modifications cannot improve functions for those with severe degrees of difficulties. The average EQ-5D-5L score had also increased by 0.203 from 0.346 at baseline to 0.549, indicating that in general the quality of life of persons with disabilities participating in the programme was improved (Table 1).

**Table 2:** Level of difficulty in performing activities, before and after home modifications, Thailand, 2013

Type and level of difficulty based on ICF code	No. (%) of persons with disabilities (*n* = 43)	
	Before home modifications	After home modifications
**Walking (d450)**		
Walk independently	1 (2.3)	3 (7.0)
Walk with abnormal gait	9 (20.9)	10 (23.3)
Walk with assistive device	14 (32.6)	10 (23.3)
Walk with assistive device and personal assistance	7 (16.3)	4 (9.3)
Cannot walk at all	12 (27.9)	16 (37.2)
**Getting up from floor (d4101)**		
Getting up independently	5 (11.6)	8 (18.6)
Getting up with assistive device	15 (34.9)	8 (18.6)
Getting up with assistive device and minimal personal assistance	2 (4.7)	2 (4.7)
Getting up with assistive device and maximal personal assistance	11 (25.6)	4 (9.3)
Cannot get up at all	10 (23.3)	21 (48.8)
**Getting up from chair or bed (d4103)**		
Getting up independently	12 (27.9)	12 (27.9)
Getting up with assistive device	11 (25.6)	14 (32.6)
Getting up with assistive device and minimal personal assistance	4 (9.3)	0 (0.0)
Getting up with assistive device and maximal personal assistance	3 (7.0)	2 (4.7)
Cannot get up at all	9 (20.9)	9 (20.9)
Not relevant	4 (9.3)	6 (14.0)
**Moving around (d460)**		
Moving independently	29 (67.4)	29 (67.4)
Moving under supervision	3 (7.0)	4 (9.3)
Moving with minimal personal assistance	2 (4.7)	3 (7.0)
Moving with maximal personal assistance	7 (16.3)	4 (9.3)
Cannot move at all	1 (2.3)	2 (4.7)
Not relevant	1 (2.3)	1 (2.3)
**Climbing (d4551)**		
Climbing independently	0 (0.0)	7 (16.3)
Climbing with personal assistance	10 (23.3)	4 (9.3)
Cannot climb at all	0 (0.0)	1 (2.3)
Not relevant	33 (76.7)	31 (72.1)
**Moving around using equipment (d465)**		
Moving independently	26 (60.5)	27 (62.8)
Moving with personal assistance	10 (23.3)	9 (20.9)
Cannot move at all	7 (16.3)	7 (16.3)

ICF: *International classification of functioning, disability and health.*

## Lessons learnt

Box 1 summarizes the main lessons learnt. The programme demonstrated that home modifications in low-resourced settings are technically and financially feasible and can lead to a reduction in functioning difficulty and improvement in the quality of life of persons with disabilities. Technical expertise for home assessment and modification design can be mobilized and supported locally, especially in areas where local training institutions, such as vocational colleges, are available. A multidisciplinary team consisting of medical and nonmedical practitioners, as well as volunteers from the community can be convened either by local government organizations or district hospitals to support a home modification programme. Local government’s ownership and leadership of the programme is critical to mobilize local resources.

Box 1 **Summary of main lessons learnt**

- An effective national policy that entitles home environment modifications for persons with disabilities needs local funding and implementation capacity to show improvement in the quality of life of persons with disabilities.
- To scale up a home environment modifications programme beyond the project site, increased government subsidies in line with the different types of modifications, additional financial and technical resources from local government and communities and technical guidelines on home modifications and training for provincial staff is needed.
- Intersectoral and multidisciplinary approach from project planning to implementation is important for successful implementation.
